# Mutant Huntingtin Does Not Affect the Intrinsic Phenotype of Human Huntington’s Disease T Lymphocytes

**DOI:** 10.1371/journal.pone.0141793

**Published:** 2015-11-03

**Authors:** James R. C. Miller, Ulrike Träger, Ralph Andre, Sarah J. Tabrizi

**Affiliations:** Department of Neurodegenerative Disease, UCL Institute of Neurology, London, United Kingdom; University of Florida, UNITED STATES

## Abstract

Huntington’s disease is a fatal neurodegenerative condition caused by a CAG repeat expansion in the huntingtin gene. The peripheral innate immune system is dysregulated in Huntington’s disease and may contribute to its pathogenesis. However, it is not clear whether or to what extent the adaptive immune system is also involved. Here, we carry out the first comprehensive investigation of human *ex vivo* T lymphocytes in Huntington’s disease, focusing on the frequency of a range of T lymphocyte subsets, as well as analysis of proliferation, cytokine production and gene transcription. In contrast to the innate immune system, the intrinsic phenotype of T lymphocytes does not appear to be affected by the presence of mutant huntingtin, with Huntington’s disease T lymphocytes exhibiting no significant functional differences compared to control cells. The transcriptional profile of T lymphocytes also does not appear to be significantly affected, suggesting that peripheral immune dysfunction in Huntington’s disease is likely to be mediated primarily by the innate rather than the adaptive immune system. This study increases our understanding of the effects of Huntington’s disease on peripheral tissues, while further demonstrating the differential effects of the mutant protein on different but related cell types. Finally, this study suggests that the potential use of novel therapeutics aimed at modulating the Huntington’s disease innate immune system should not be extended to include the adaptive immune system.

## Introduction

Huntington’s disease (HD) is a fatal, autosomal dominant neurodegenerative condition caused by a CAG repeat expansion in exon 1 of the huntingtin (*HTT*) gene on chromosome 4 [1]. A sequence of 40 or more CAG repeats is fully penetrant and results in the disease. Patients are affected by a combination of symptoms, including movement disorders and cognitive impairment, and the disease is uniformly fatal 15–20 years after its onset [2]. No treatments are currently available to slow the progression of the disease. Disease pathology has been traditionally considered to be confined to the central nervous system (CNS), but in recent years it has become apparent that HD is a disease of the whole body, with patients being affected by peripheral pathology including skeletal muscle wasting and testicular atrophy [3]. Indeed, the mutant (m)HTT protein has been found to be expressed in all cells and tissues that have been studied [4].

Immune system dysfunction is a recognised part of HD pathogenesis, both in the CNS in the form of microglial activation [5], and the periphery where increased circulating levels of pro-inflammatory cytokines [6] and chemokines [7] are detectable in the blood many years before the onset of disease. The peripheral immune system may act as a modifier of disease progression, with the administration of immunomodulatory drugs improving the phenotype of HD mouse models [8, 9]. Recent work attempting to identify the cause(s) of this dysfunction has focused on the cells of the innate immune system. Indeed, HD myeloid cells are hyper-reactive in response to lipopolysaccharide (LPS), producing significantly elevated levels of pro-inflammatory cytokines such as IL-6 and TNFα [10]. This is due at least in part to a direct interaction of the mHTT protein with IKK, the key kinase in the NFκB pathway, and these changes are reversible following HTT lowering with small interfering RNA (siRNA). HD myeloid cells have also been shown to exhibit functional deficits when migrating to chemotactic stimuli [11] and phagocytosing fluorescent beads [12].

However, very little is known about the adaptive immune system in HD. In contrast to the innate immune system, which provides an immediate generic response to a wide range of pathogens, the adaptive immune system generates a highly specific secondary response that is also known as immunological memory. The adaptive immune system is largely mediated by T lymphocytes, which produce cytokines to enhance or suppress the activity of other immune cells, and B lymphocytes that generate antibodies following exposure to specific pathogens. T lymphocytes are further categorised as a number of subsets, including CD4^+^ helper T lymphocytes (including T_h_1 and T_h_2 lymphocytes) and CD8^+^ cytotoxic T lymphocytes. There is much interplay between the innate and adaptive immune systems, which is required for both to function effectively [13, 14]. For example, professional antigen presenting cells such as dendritic cells and macrophages display exogenous antigens on major histocompatibility complex (MHC) class II molecules in order to activate CD4^+^ helper T lymphocytes, which respond by differentiating and producing a range of cytokines that direct both the innate and adaptive immune responses [15]. It is not clear currently whether such interplay is affected in HD, or whether HD immune dysfunction is universal or restricted to specific cell types.

Previous work has shown that mHTT is expressed in T lymphocytes, and levels of the mutant protein in this cell type have been found to correlate with disease burden scores and caudate atrophy [16]. Levels of the signature T_h_2 cytokine IL-4 are also significantly elevated in the plasma of HD patients [6], suggesting that mHTT may have an adverse effect on T lymphocyte function. In this study we carry out the first comprehensive investigation of human *ex vivo* HD T lymphocytes, with a focus on CD4^+^ helper T lymphocytes (one of the main effector cells of the adaptive immune system). The frequency of a range of T lymphocyte subsets was measured by flow cytometry, before the intrinsic function of HD T lymphocytes was examined using proliferation assays and cytokine profiling. Finally, the expression levels of 84 key T lymphocyte related genes were investigated using quantitative polymerase chain reaction (qPCR) arrays. These data have important implications for potential future therapeutic strategies aimed at modulating the immune system in HD.

## Materials and Methods

### Collection and classification of human samples

All human experiments were performed in accordance with the Declaration of Helsinki and approved by the University College London (UCL)/UCL Hospitals Joint Research Ethics Committee. Blood samples were donated by genetically-diagnosed HD patients and control subjects, and all subjects provided informed written consent. HD subjects were classified on the basis of their total functional capacity (TFC) score; only manifest subjects with early or moderate stage disease (TFC 13–3) were included in the study. The HD and control groups were age-matched for all experiments (S1 Table). Subjects with inflammatory or infective conditions were excluded from the study.

### Isolation of peripheral blood mononuclear cells

Peripheral blood mononuclear cells (PBMCs) were isolated from human peripheral blood samples by density centrifugation. Twenty five ml peripheral blood was layered on top of 20 ml Histopaque-1077 (Sigma-Aldrich) in 50 ml tubes, before centrifuging at 400 xg for 30 min with minimum deceleration. The PBMC layer was then transferred to a fresh tube using a Pasteur pipette.

### Cell culture and stimulation

Cells were cultured in RPMI 1640 culture medium supplemented with 10% foetal bovine serum (FBS), 2 mM L-glutamine, 50 U/ml penicillin and 50 μg/ml streptomycin (Invitrogen). CD3 stimulation was carried out using plate-bound functional grade anti-human CD3 antibody (eBioscience). Five μg/ml antibody diluted in PBS was added to each culture well before incubating at 4°C for 24 h. The wells were washed with PBS to remove any unbound antibody, before 2 μg/ml functional grade anti-human CD28 antibody (eBioscience) was added to the culture medium to provide appropriate co-stimulation. PHA-P stimulation was carried out by adding 10 μg/ml PHA-P (Sigma-Aldrich) directly to the culture medium.

### Flow cytometry

A maximum of 1 x 10^6^ cells were transferred into a V-bottomed 96 well plate and centrifuged at 400 xg for 5 min. Staining of cell surface markers was carried out by resuspending the cells in 50 μl antibody suitably diluted in FACS buffer (PBS with 1% FBS and 0.02% sodium azide). A full list of antibodies used is included in S2 Table. Cells were stained for 60 min on a shaker at 4°C in the dark before washing in 200 μl FACS buffer by centrifuging at 400 xg for 5 min. Cells were then fixed using 4% paraformaldehyde in PBS for 10 min. After fixing the cells were washed again and transferred to FACS tubes for analysis. Flow cytometry was carried out using a MACSQuant flow cytometer (Miltenyi Biotech) with MACSQuantify software. Data analysis was performed using FlowJo 7.2.5 (Tree Star) software. Non-stained and single colour stained controls were always performed.

### T lymphocyte proliferation assays

Cell staining for proliferation assays was carried out using carboxyfluorescein succinimidyl ester (CFSE). After isolation PBMCs were counted using a Neubauer counting chamber and resuspended at 1 x 10^7^ cells per ml of PBS with 5% FBS to buffer against the toxic effects of CFSE. CFSE was added to the suspension for a final concentration of 5 μM, before the sample was mixed vigorously and incubated for 10 min at room temperature in the dark. Labelling was stopped by adding cold RPMI medium with 10% FBS and incubating on ice for 5 min. After labelling the cells were seeded at 5 x 10^5^ cells per well in 48 well plates, and were either left unstimulated or stimulated with anti-CD3 and anti-CD28 antibodies or PHA-P as described above. Unstained cells were also seeded to provide appropriate controls for flow cytometry. Cells were harvested after 72, 96 and 120 h and stained with Fixable Viability Dye 660 (eBioscience) according to the manufacturer’s instructions. Cells were then stained and analysed by flow cytometry as described above.

### Magnetic cell sorting

CD4^+^ T lymphocytes were isolated by magnetic cell sorting. PBMCs were counted using a Neubauer counting chamber before being resuspended in 10 μl anti-human CD4 beads (Miltenyi Biotec) and 90 μl MACS buffer (PBS with 1% BSA and 2 mM EDTA) per 10^7^ cells. After 15 min incubation at 4°C the samples were centrifuged at 400 xg for 5 min before being resuspended in 500 μl MACS buffer. Magnetic cell sorting was carried out by placing MACS columns (Miltenyi Biotec) in a magnetic field and adding each cell suspension to an individual column. The columns were washed with MACS buffer before magnetically labelled CD4^+^ T lymphocytes were eluted by taking the columns out of the magnetic field and plunging MACS buffer through the column. On average, the sorted cell populations contained 85–95% CD4^+^ cells.

### Cytokine profiling

CD4^+^ T lymphocytes were isolated as described above, before being seeded at 1 x 10^5^ cells per well in 96 well plates. Cells were either left unstimulated or stimulated with anti-CD3 and anti-CD28 antibodies for 48 h as described above. After 48 h the supernatants were collected and analysed using V-PLEX Pro-inflammatory Panel 1 (human) and V-PLEX Human IL-5 kits (Meso Scale Discovery) according to the manufacturer’s instructions. Data analysis was carried out using Discovery Workbench 4.0 (Meso Scale Discovery), before cytokine values were normalised to the total protein content of the well. Protein values were measured using the Pierce bicinchoninic acid (BCA) protein assay kit (Thermo Scientific) according to the manufacturer’s instructions.

### Quantitative polymerase chain reaction arrays

CD4^+^ T lymphocytes were isolated as described above, prior to seeding at 5 x 10^6^ cells per dish in 60 mm dishes. Cells were either left unstimulated or stimulated with anti-CD3 and anti-CD28 antibodies for 8 h as described above. After 8 h the cells were harvested and RNA was extracted using the RNeasy Mini Kit (Qiagen) according to the manufacturer’s instructions. The concentration of each sample was measured using an ND-1000 Spectrophotometer (NanoDrop), before RNA integrity was assessed using 2100 Bioanalyser NanoChips (Agilent). Only samples with non-degraded RNA were used for the quantitative polymerase chain reaction (qPCR) arrays.

Six hundred ng of RNA from each sample was reverse transcribed using the RT^2^ First Strand Kit (Qiagen) and analysed using the Human T_h_1 and T_h_2 Responses RT^2^ Profiler PCR Array (SABiosciences) according to the manufacturers’ instructions. SYBR green was used as the reporter dye while ROX was used as the passive dye. Prior to analysis a consistent threshold value was set for each sample and any samples showing signs of genomic DNA contamination were excluded. Data analysis was carried out using the online data analysis tool provided by the manufacturer (http://pcrdataanalysis.sabiosciences.com/pcr/arrayanalysis.php). Genes with an average C_t_ value of >35 were excluded from the final results.

### Statistical analysis

Statistical analysis of immune cell subset, proliferation and cytokine profiling data was carried out using GraphPad Prism 6 (GraphPad), while qPCR array data was analysed using the manufacturer’s online tool as outlined above. All experiments were analysed using unpaired two-tailed Student’s *t*-tests. Where appropriate post-hoc analysis of multiple t tests was carried out using a Holm-Šídák correction (alpha = 0.05) for multiple comparisons. All error bars represent standard error of the mean.

## Results

### The frequency of T lymphocyte subsets does not differ between HD and control peripheral blood

**Fig 1:**
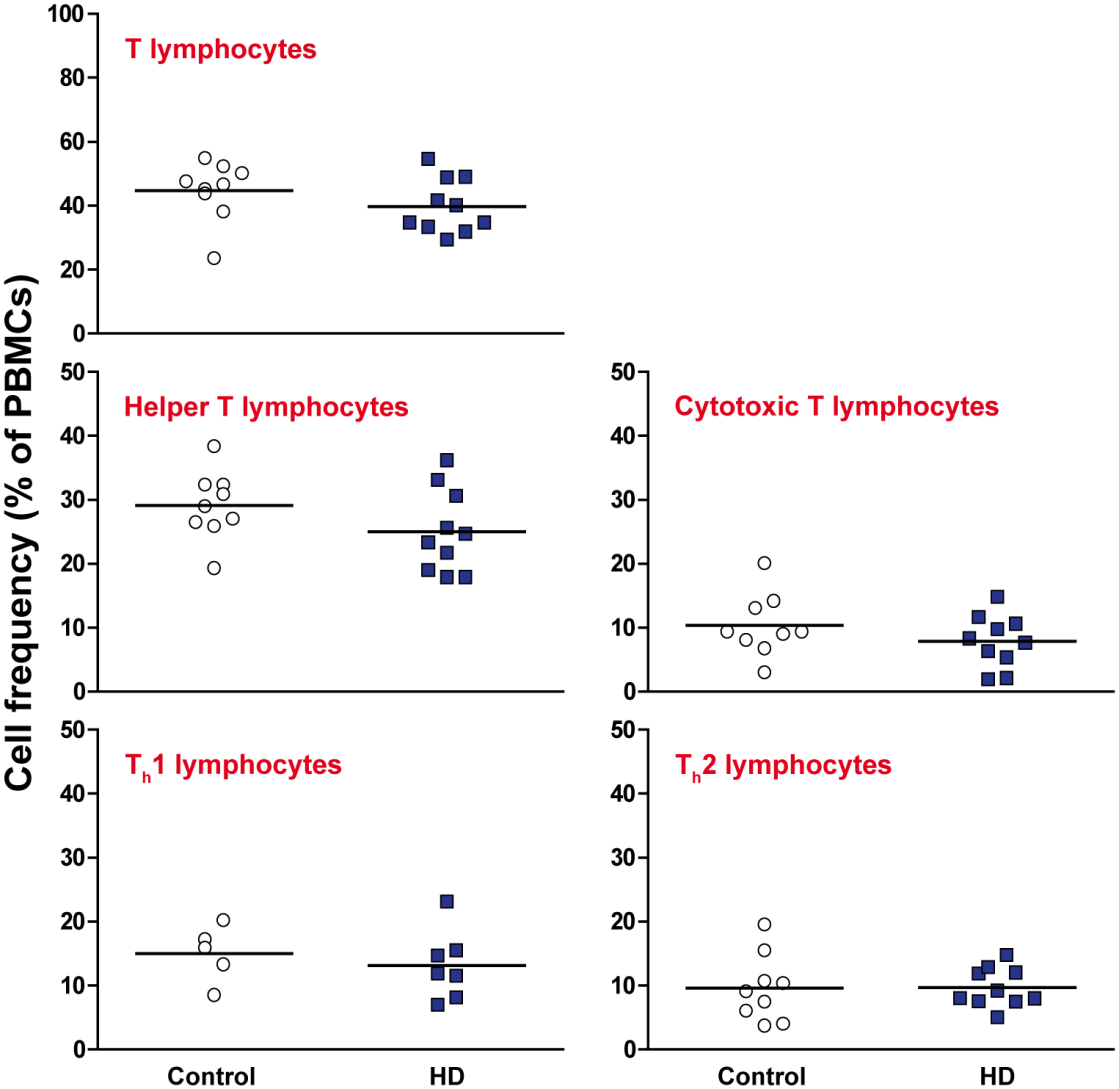
The frequency of T lymphocyte subsets does not differ between HD and control peripheral blood. The frequencies of a range of T lymphocyte subsets in the isolated PBMC populations were measured by flow cytometry using antibodies to CD3^+^ T lymphocytes, CD3^+^ CD4^+^ helper T lymphocytes and CD3^+^ CD8^+^ cytotoxic T lymphocytes. Within the CD3^+^ CD4^+^ helper T lymphocyte population, the T_h_1 and T_h_2 subtypes were analysed using antibodies to CXCR3 and CCR4 respectively. No significant differences were seen between HD and control for any of the cell types that were analysed. Statistical analysis was carried out using two-tailed unpaired student’s t tests.

**Fig 2:**
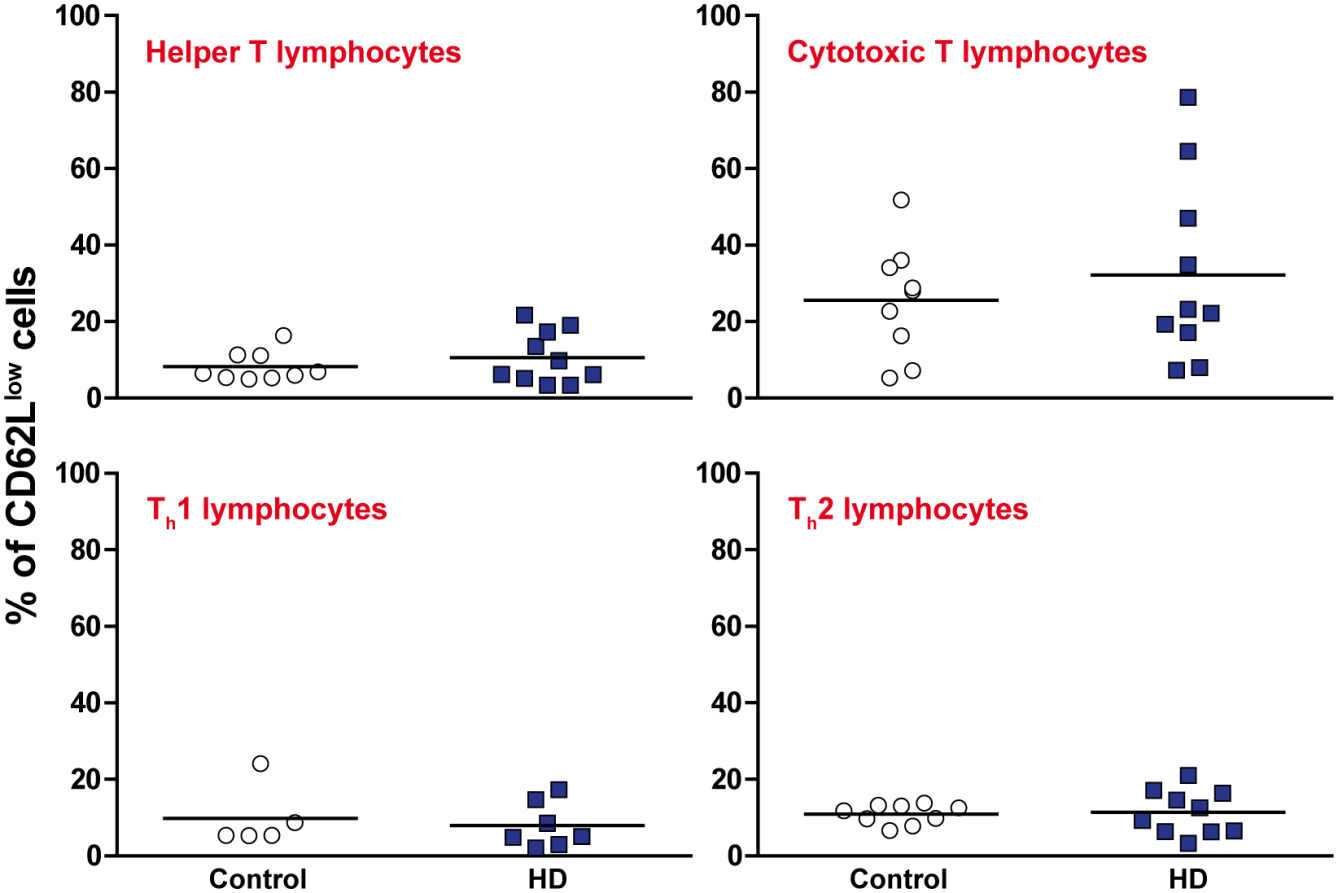
Activation levels of T lymphocyte subsets are not altered between HD and control peripheral blood. Within the previously analysed T lymphocyte populations (Fig 1), the activation levels of each were measured using CD62L^low^ expression. No significant differences were seen in the activation levels of any of the HD populations compared to control. Statistical analysis was carried out using two-tailed unpaired student’s t tests.

It has been demonstrated previously that circulating levels of pro-inflammatory cytokines and chemokines are elevated in HD plasma [6, 7]. One possible cause of this is a shift in the relative proportions of different immune cell subtypes, leading to changes in the overall cytokine environment being produced. PBMCs were isolated from manifest HD and control blood samples and stained with antibodies for CD3^+^ T lymphocytes, CD3^+^ CD4^+^ helper T lymphocytes and CD3^+^ CD8^+^ cytotoxic T lymphocytes. Helper T lymphocytes were further investigated using antibodies for CXCR3^+^ T_h_1 lymphocytes and CCR4^+^ T_h_2 lymphocytes. No significant differences were detected for any of the T lymphocyte subtypes measured as a percentage of the HD and control PBMC populations (Fig 1, gating schematic in S1 Fig). This is consistent with a recent study that showed that the frequency of CD3^+^ T lymphocytes is not altered in the spleen and bone marrow of R6/2 and wild-type mice [12]. The activation levels of CD4^+^ and CD8^+^ T lymphocytes were also assessed by CD62L^low^ expression. Again, no significant differences were seen between the HD and control PBMC populations (Fig 2).

### T lymphocyte proliferation in response to stimuli is not impaired in HD compared to control

HD myeloid cells display functional deficits when migrating to chemotactic stimuli and phagocytosing fluorescent beads [11, 12]. Here, we sought to investigate whether HD T lymphocytes exhibit similar functional abnormalities. The ability of lymphocytes to rapidly proliferate in response to a stimulus is a key feature of the adaptive immune system. PBMCs were isolated from manifest HD and control peripheral blood samples before being stained with the intracellular dye CFSE. CFSE is a highly stable fluorescent dye that is evenly divided between the two daughter cells produced when a cell divides. This allows the tracking of multiple cell divisions *in vitro* by flow cytometry as a sequential halving of fluorescence intensity [17, 18]. After staining, the PBMC populations were either left unstimulated or stimulated with either anti-CD3 and anti-CD28 antibodies or PHA-P. This enabled us test the cells’ response to antigenic and mitogenic stimuli, respectively. Cultures were harvested after 72, 96 and 120 h to build up a broad profile of the proliferative response.

**Fig 3:**
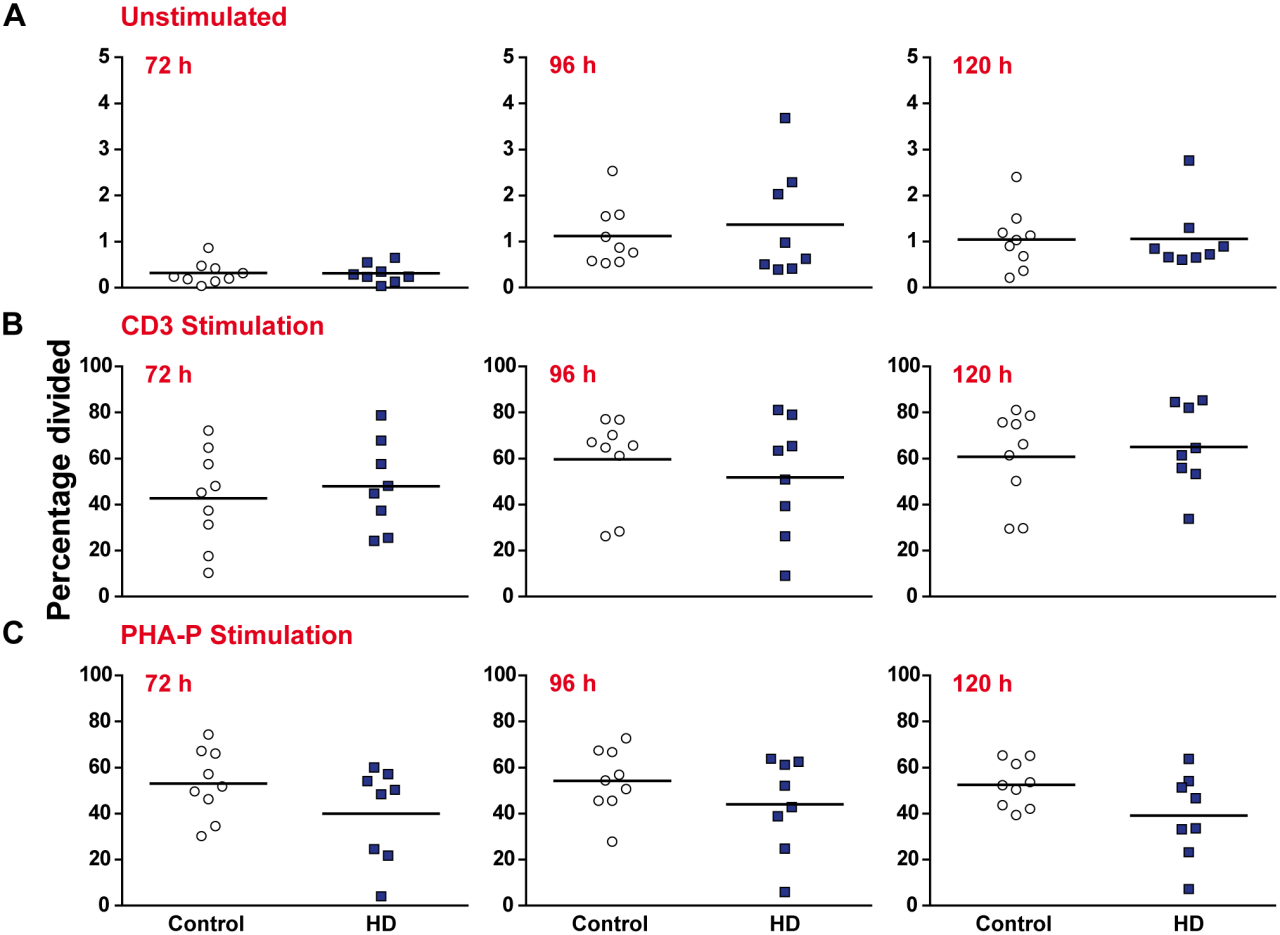
The percentage of proliferating CD3^+^ T lymphocytes is not affected in HD compared to control. PBMCs were stained with CFSE and the percentage of CD3^+^ T lymphocytes from the initial culture which divided **(A)** in the absence of stimulation **(B)** with 5 μg/ml anti-CD3 and 2 μg/ml anti-CD28 antibody stimulation and **(C)** with 10 μg/ml PHA-P stimulation was measured after 72, 96 and 120 h by flow cytometry. No significant differences were seen between HD and control for any of the experimental time points or conditions. Statistical analysis was carried out using two-tailed unpaired student’s t tests with a Holm-Šídák correction (alpha = 0.05) for multiple comparisons.

**Fig 4:**
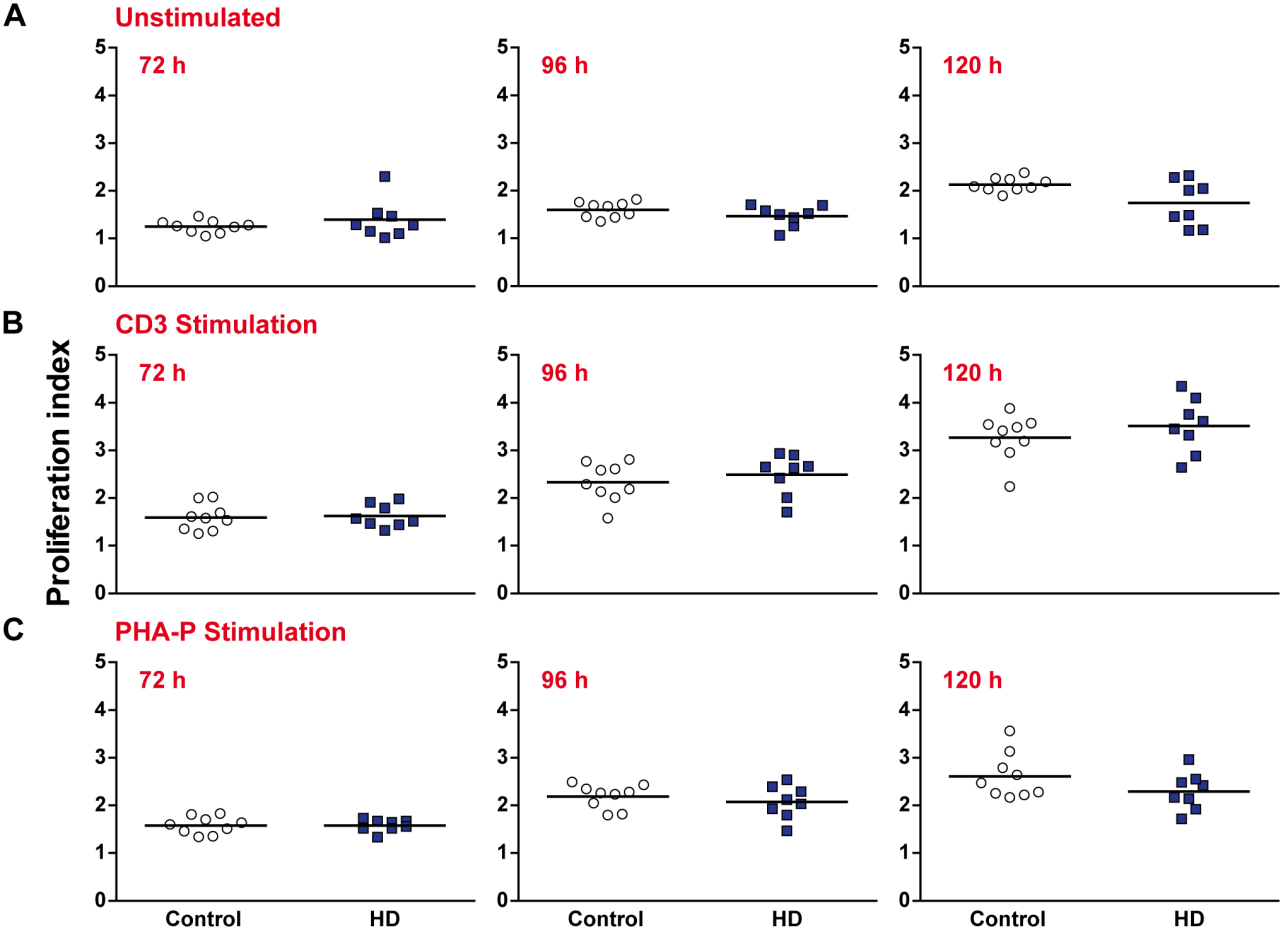
The number of divisions which proliferating CD3^+^ T lymphocytes undergo is not affected in HD. PBMCs were stained with the intracellular dye CFSE and the number of divisions which proliferating CD3^+^ T lymphocytes underwent **(A)** in the absence of stimulation **(B)** with 5 μg/ml anti-CD3 and 2 μg/ml anti-CD28 antibody stimulation and **(C)** with 10 μg/ml PHA-P stimulation was measured after 72, 96 and 120 h by flow cytometry. No significant differences were seen between HD and control for any of the experimental time points or conditions. Statistical analysis was carried out using two-tailed unpaired student’s t tests with a Holm-Šídák correction (alpha = 0.05) for multiple comparisons.

The proliferative response of three T lymphocyte populations (all CD3^+^, CD3^+^ CD4^+^ and CD3^+^ CD8^+^ cells, gating schematic in S2 Fig) was analysed using a range of statistics as outlined by Roederer [19]. No significant differences were seen in the percentage of CD3^+^ T lymphocytes from the initial culture that entered cell division (percentage divided) for any of the experimental conditions or time points (Fig 3). Similarly, no significant differences were seen in the average number of divisions each dividing CD3^+^ T lymphocyte underwent (proliferation index) (Fig 4). Similar results were obtained when the CD4^+^ and CD8^+^ subtypes were analysed, with no significant differences being observed for any of the experimental conditions or time points (S3 and S4 Tables). Additional analysis was carried out to determine the average number of divisions all cells in culture underwent (division index) (S5 Table), as well as the percentage of cells in the final culture which had divided at least once (fraction diluted) (S6 Table). Again, no significant differences were seen between the HD and control samples. These results demonstrate that proliferation in response to a stimulus is not impaired in any of the major T lymphocyte populations in HD.

### Cytokine production by *ex vivo* HD helper T lymphocytes is not altered compared to controls

HD monocytes and macrophages are hyper-reactive and produce significantly elevated levels of IL-6 and TNFα following LPS stimulation compared to control cells [10]. Here, we investigated whether HD T lymphocytes have a similarly hyper-reactive phenotype in response to a stimulus. CD4^+^ helper T lymphocytes were isolated from manifest HD and control peripheral blood samples and stimulated with anti-CD3 and anti-CD28 antibodies for 48 h, after which the cell culture supernatants were collected and analysed using multiplex assays. Unstimulated cells were also seeded in order to investigate baseline cytokine levels.

**Fig 5:**
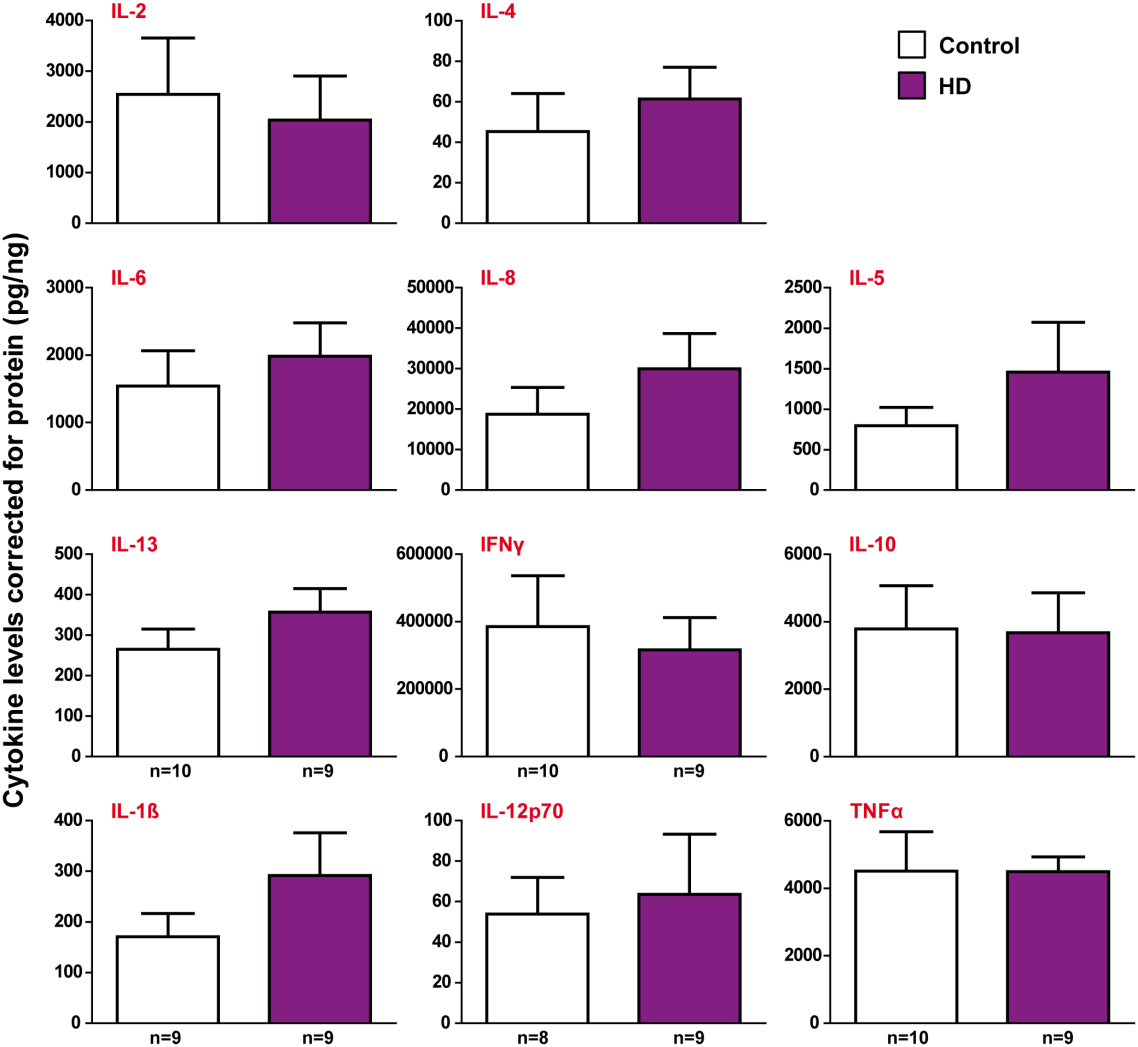
Cytokine production by stimulated HD helper T lymphocytes is not altered compared to controls. CD4^+^ helper T lymphocytes were isolated from HD and control peripheral blood using magnetic cell sorting and stimulated with 5 μg/ml anti-CD3 and 2 μg/ml anti-CD28 antibodies. Supernatants were collected after 48 h and cytokine profiling was carried out. No significant differences were seen in the levels of any of the cytokines produced by HD and control CD4^+^ T lymphocytes after normalisation to total protein levels. Data show mean concentrations ± SEM. Statistical analysis was carried out using two-tailed unpaired student’s t tests.

In contrast with HD myeloid cells, no significant differences were seen in the levels of any of the eleven cytokines that were measured in the stimulated HD and control samples (Fig 5). A number of cytokines in the unstimulated samples were below the detection limits of the assay, but those which were measurable also showed no significant differences between the HD and control samples (S3 Fig). These results demonstrate that the cytokine profile of HD helper T lymphocytes is not significantly affected by the presence of mHTT.

### The transcriptional profile of HD helper T lymphocytes is not dysregulated compared to controls

While these experiments have suggested that the intrinsic T lymphocyte phenotype is not altered between HD and control subjects, it is still possible that there are transcriptional differences that do not have a detectable effect at the functional level in an *ex vivo* setting. Transcriptional dysregulation is a key feature of HD pathogenesis [20], whereby the mHTT protein sequesters transcription factors [21] and binds directly to DNA [22]. Transcriptional changes have previously been demonstrated in HD myeloid cells, with reversal of IRAK1, CD40 and JUN expression differences being achieved following HTT lowering with siRNA [10]. Here, we sought to investigate whether the mHTT protein has a similar effect on the transcriptional profiles of HD T lymphocytes. CD4^+^ helper T lymphocytes were isolated from manifest HD and control peripheral blood samples and either left unstimulated or stimulated with anti-CD3 and anti-CD28 antibodies for 8 h. The messenger RNA (mRNA) expression of eighty four key T lymphocyte related genes was then measured using the SABiosciences Human T_h_1 and T_h_2 Responses RT^2^ Profiler PCR Array.

We identified two genes which were significantly upregulated (p<0.05) in the unstimulated HD samples compared to control (TNFSF4, PTGDR2) (Table 1), and three genes which were significantly upregulated in the stimulated HD samples compared to control (TYK2, TNFRSF8, STAT6) (Table 2). No genes were found to be significantly downregulated in either the unstimulated or stimulated HD samples compared to control. However, none of the upregulated genes had a fold change of >1.8 between HD and control, and only two (PTGDR2, TNFRSF8) had a fold change of >1.5. Furthermore, the expression of signalling molecules such as MAPK8 (JNK1) and JAK1/2 was not altered in either the unstimulated or stimulated HD samples (complete data in S7 and S8 Tables). Consistent with the cytokine profiling data, the mRNA levels of IFNγ, IL-2, IL-5, IL-6, IL-10, IL-13 and TNFα were not altered between HD and control in either the stimulated or unstimulated samples. IL-4 and IL12B transcript levels were not detectable in the unstimulated samples, but were similarly unchanged in the stimulated samples. IL-1β and IL-8 were not included in the list of genes measured by the array. The lack of IL-10 expression changes are a further contrast with HD myeloid cells, as unstimulated HD monocytes were found to express significantly more IL-10 mRNA than controls [10]. Taken together, these results suggest that sub-phenotypic transcriptional dysregulation is not a significant phenomenon in HD helper T lymphocytes.

**Table 1:** The top ten gene changes in unstimulated Huntington’s disease helper T lymphocytes compared to controls.

Gene name	Fold change	P value
TNFSF4	1.471	0.008
PTGDR2	1.788	0.014
GATA3	1.177	0.056
IL18R1	1.336	0.058
IL5	2.245	0.063
LAT	0.893	0.101
IL2RA	1.154	0.102
CSF2	0.490	0.124
JAK1	1.108	0.137
CCR4	1.258	0.183

Data presented as fold change calculated from ΔΔ-CT values, unpaired two-tailed t-test used as statistical method.

**Table 2:** The top ten gene changes in stimulated Huntington’s disease helper T lymphocytes compared to controls.

Gene name	Fold change	p value
TYK2	1.176	0.002
TNFRSF8	1.702	0.013
STAT6	1.231	0.035
TNFSF4	1.756	0.066
JAK1	1.169	0.072
CCR4	1.191	0.118
IL18R1	1.129	0.149
CD27	1.261	0.152
STAT1	0.844	0.162
IL15	0.800	0.184

Cells were stimulated with anti-CD3 and anti-CD28 antibodies for 8 h prior to analysis. Data presented as fold change calculated from ΔΔ-CT values, unpaired two-tailed t-test used as statistical method.

## Discussion

Peripheral immune dysfunction is an established feature of HD pathogenesis, with elevated plasma cytokine and chemokine levels being detectable up to 16 years before the predicted onset of disease [6, 7]. Recent work has focused on identifying the cause of this dysfunction, with HD monocytes and macrophages being found to produce significantly increased levels of pro-inflammatory molecules in a HTT-dependent manner following LPS stimulation [10]. However, this does not preclude other cell types from playing a contributing role in HD peripheral immune dysfunction. T lymphocytes are vital for adaptive immunity, performing a wide range of effector and regulatory functions in the body’s defence against pathogens. The close interplay between T lymphocytes and myeloid cells, in the form of antigen presentation and cytokine feedback loops, means that the investigation of T lymphocytes is important in determining whether HD immune dysfunction is universal or restricted to specific cell types.

Similarly to myeloid cells in HD, T lymphocytes express mHTT, the levels of which have been found to correlate with disease burden scores and caudate atrophy in HD patients [16]. While the presence of mHTT does not guarantee phenotypic dysfunction, plasma levels of the signature T_h_2 cytokine IL-4 are elevated in manifest HD patients [6]. However, the same study also found that immunoglobulin (Ig) levels were not altered between HD patients and controls, pointing away from any substantial widespread dysregulation of the adaptive immune system. T lymphocytes were also found not to be enriched in post-mortem HD brain tissue [23], suggesting that they may not play a key role in CNS inflammation in HD. In order to answer the question of whether the peripheral adaptive immune system is contributing to HD pathogenesis, we undertook a comprehensive, multi-faceted approach to investigating whether the intrinsic biology of T lymphocytes is affected in HD.

One potential cause of immune dysfunction is a shift in the relative frequencies of immune cell subsets, resulting in a knock on effect on the overall cytokine and chemokine environment. Here we show that the proportions of the main T lymphocyte subsets are not altered in HD and control peripheral blood samples. This is consistent with a previous study showing that the frequency of CD3^+^ T lymphocytes is not altered in the spleen and bone marrow of R6/2 and wild-type mice [12]. Taken together, these studies point away from any widespread changes in HD immune cell frequencies, and suggest that any immune alterations are likely to be the result of functional differences and not differences in the relative numbers of different cell types. Furthermore, we have shown that the activation levels of T lymphocytes as measured by CD62L^low^ in HD are no different to controls, suggesting that they do not display significantly more activation in their normal physiological state.

In addition in their hyper-reactive cytokine profiles, HD myeloid cells have been shown to be functionally abnormal in tests of migratory and phagocytic capability [11, 12]. Here we show that HD T lymphocytes do not display similar functional deficits when proliferating in response to a stimulus. This is the case throughout the proliferative response, and applies to both the percentage of dividing cells and the number of divisions those cells undergo. In addition, CD4^+^ helper T lymphocytes had a similar cytokine profile to controls following antigenic stimulation. These results suggest that, unlike HD myeloid cells, HD T lymphocytes do not suffer from any intrinsic functional impairment. One possible explanation for this contrast is the relative contributions of different intracellular signalling pathways.

It has previously been shown that mHTT directly interacts with IKK, the key kinase in the NFĸB pathway [24]. This leads to an increased translocation of NFĸB to the nucleus and an up-regulation of NFĸB related genes [10]. In myeloid cells the NFĸB pathway is vital for the production of pro-inflammatory cytokines such as IL-6 and TNFα [25], and NFĸB dysfunction in HD myeloid cells is likely to be a key reason for their hyper-reactive phenotype. While the NFĸB pathway also plays an important role in T lymphocyte signalling [25], additional transcription factors such as the NFAT and MAPK families are heavily involved in processes such as cytokine secretion [26, 27]. It is therefore possible that while similar NFĸB dysfunction may exist in HD T lymphocytes, a relatively reduced role of the pathway means compensatory mechanisms are in place to prevent obvious phenotypic dysfunction. The mechanism of NFĸB activation is also different in each cell type. NFĸB activation in myeloid cells takes place as a result of LPS stimulation of the TLR4 receptor [28], whereas T lymphocytes are classically stimulated via the T lymphocyte receptor [29]. While TLR4 is expressed by helper T lymphocytes, its exact function remains unclear, and has even been suggested to repress T lymphocyte activation [30]. It has also been suggested that NFĸB has anti-inflammatory functions in naïve CD4^+^ T lymphocytes, with overexpression of the p50 subunit of NFĸB leading to reduced transcription of the signature T_h_1 cytokine IL-2 [31]. Taken together, these results suggest that the lack of obvious phenotypic changes may be due to the altered and reduced role of NFĸB signalling in helper T lymphocytes compared to myeloid cells.

Transcriptional dysregulation is a key feature of HD pathogenesis [20], with mHTT aggregates thought to sequester transcription factors [21] in addition to mHTT fragments binding directly to DNA [22]. However, the effect of mHTT is likely to vary between different signalling pathways. Here we demonstrate that widespread changes in T lymphocyte related genes are lacking in HD helper T lymphocytes in both the unstimulated and stimulated states. Key signalling molecules such as MAPK8 (JNK1) and JAK1/2 were unaffected in HD T lymphocytes, supporting our hypothesis that alternative signalling pathways are able to compensate for potential NFĸB pathway dysfunction. While the key T_h_2 transcription factor STAT6 was found to be upregulated in stimulated HD cells, no changes were found in any related molecules. Indeed, it has been shown that JAK/STAT signalling is not significantly affected in HD myeloid cells [32], making it likely that this result (fold change 1.23) does not have a great deal of functional significance. Furthermore, the transcript levels of pro-inflammatory cytokines such as IL-6 and TNFα are unchanged between HD and control cells. Interestingly, we have previously shown that IL-10 mRNA is significantly upregulated in HD myeloid cells compared to controls [10], but no such difference exists in helper T lymphocytes. These results support our conclusion that mHTT does not affect the intrinsic phenotype of HD helper T lymphocytes in the same way it affects HD myeloid cells.

It is important to note that these results do not exclude the possibility of differences in T lymphocyte function *in vivo*, as excessive stimulation from hyper-reactive myeloid cells could provoke increased cytokine release from T lymphocytes through feedback loops. This is one potential explanation for the increased IL-4 levels seen in manifest HD plasma [6], although it has also been suggested that increased secretion of this anti-inflammatory cytokine may be a compensatory response to chronic inflammation [33]. This conclusion is supported by the increased IL-4 levels only being seen in the later stages of the disease. However, such a complicated functional experiment is beyond the scope of this study.

Here we have shown that the intrinsic phenotype of helper T lymphocytes is not significantly affected by the presence of mHTT. This advances our understanding of HD as a disease of the whole body, and supports our previous work suggesting that hyper-reactive monocytes and macrophages are the most likely source of peripheral immune dysfunction in HD [10]. This has important implications for future novel therapies aimed at modulating the peripheral immune system, as it shows that only targeting certain cell types is likely to be beneficial. Furthermore, it demonstrates the value of a systematic approach to investigating the function of different cell types in HD, as the phenotypic effects of mHTT are clearly dependent on the cellular environment and the relative contributions of the pathways it interacts with.

## Supporting Information

S1 Fig Gating strategy for T lymphocyte subset analysis. After gating on live cells using a FSC/SSC gate, a CD3 vs. FSC plot was used to determine the percentage of CD3^+^ T lymphocytes. Within the CD3^+^ population, the percentages of CD4^+^ helper T lymphocytes and CD8^+^ cytotoxic T lymphocytes were determined, before the percentages of CXCR3^+^ T_h_1 and CCR4^+^ T_h_2 lymphocytes within the CD4^+^ population were determined. Activation levels of T lymphocytes based on CD62L^low^ expression were also determined.
(TIF)

S2 Fig Gating strategy for T lymphocyte proliferation analysis. After gating on live cells using a FSC/SSC gate, non-viable cells were excluded using Fixable Viability Dye 660. A CD3 vs. FSC plot was then used to determine the percentage of CD3^+^ T lymphocytes. Within the CD3^+^ population, the percentages of CD4^+^ helper T lymphocytes and CD8^+^ cytotoxic T lymphocytes were determined before a histogram was used to analyse the CFSE fluorescence profile of each cell population. The fraction diluted statistic was calculated by creating a univariate gate below the undivided peak, while all other proliferation statistics were calculated using the proliferation analysis tool included in the FlowJo software.
(TIF)

S3 Fig Cytokine production by unstimulated CD4^+^ helper T lymphocytes is not affected in HD vs. control. CD4^+^ helper T lymphocytes were isolated from HD and control peripheral blood using magnetic cell sorting and seeded without stimulation. Supernatants were collected after 48 h and cytokine profiling was carried out. No significant differences were seen in the detectable levels of any cytokines produced by HD and control CD4^+^ T lymphocytes after normalisation to total protein levels. Data show mean concentrations ± SEM. Statistical analysis was carried out using two-tailed unpaired student’s t tests.
(TIF)

S1 Table Age and *HTT* CAG repeat lengths of subjects participating in the study. The cohort used for each experiment is listed separately.
(DOCX)

S2 Table Directly conjugated antibodies used for flow cytometry. (DOCX)

S3 Table Analysis of T lymphocyte proliferation data using the percentage divided statistic. Percentage divided is calculated as the percentage of cells from the initial culture which divided at least once. HD n = 8, control n = 9. Data shown as mean ± SEM.
(DOCX)

S4 Table Analysis of T lymphocyte proliferation data using the proliferation index statistic. Proliferation index is calculated as the average number of divisions undergone by the dividing cells only. HD n = 8, control n = 9. Data shown as mean ± SEM.
(DOCX)

S5 Table Analysis of T lymphocyte proliferation data using the division index statistic. Division index is calculated as the average number of divisions undergone by all cells in culture. HD n = 8, control n = 9. Data shown as mean ± SEM.
(DOCX)

S6 Table Analysis of T lymphocyte proliferation data using the fraction diluted statistic. Fraction diluted is calculated as the percentage of cells in the final culture which have divided at least once. HD n = 8, control n = 9. Data shown as mean ± SEM.
(DOCX)

S7 Table Complete list of expression changes observed in unstimulated HD helper T lymphocytes compared to controls. Data presented as fold change calculated from ΔΔ-CT values, unpaired two-tailed t-test used as statistical method.
(DOCX)

S8 Table Complete list of expression changes observed in stimulated HD helper T lymphocytes compared to controls. Data presented as fold change calculated from ΔΔ-CT values, unpaired two-tailed t-test used as statistical method.
(DOCX)
