# Supplementary figures and images for: Mutant Huntingtin Does Not Affect the Intrinsic Phenotype of Human Huntington’s Disease T Lymphocytes
Source: PLoS One. 2015 Nov 3;10(11):e0141793. doi: 10.1371/journal.pone.0141793 (PMC4631523; doi:10.1371/journal.pone.0141793)

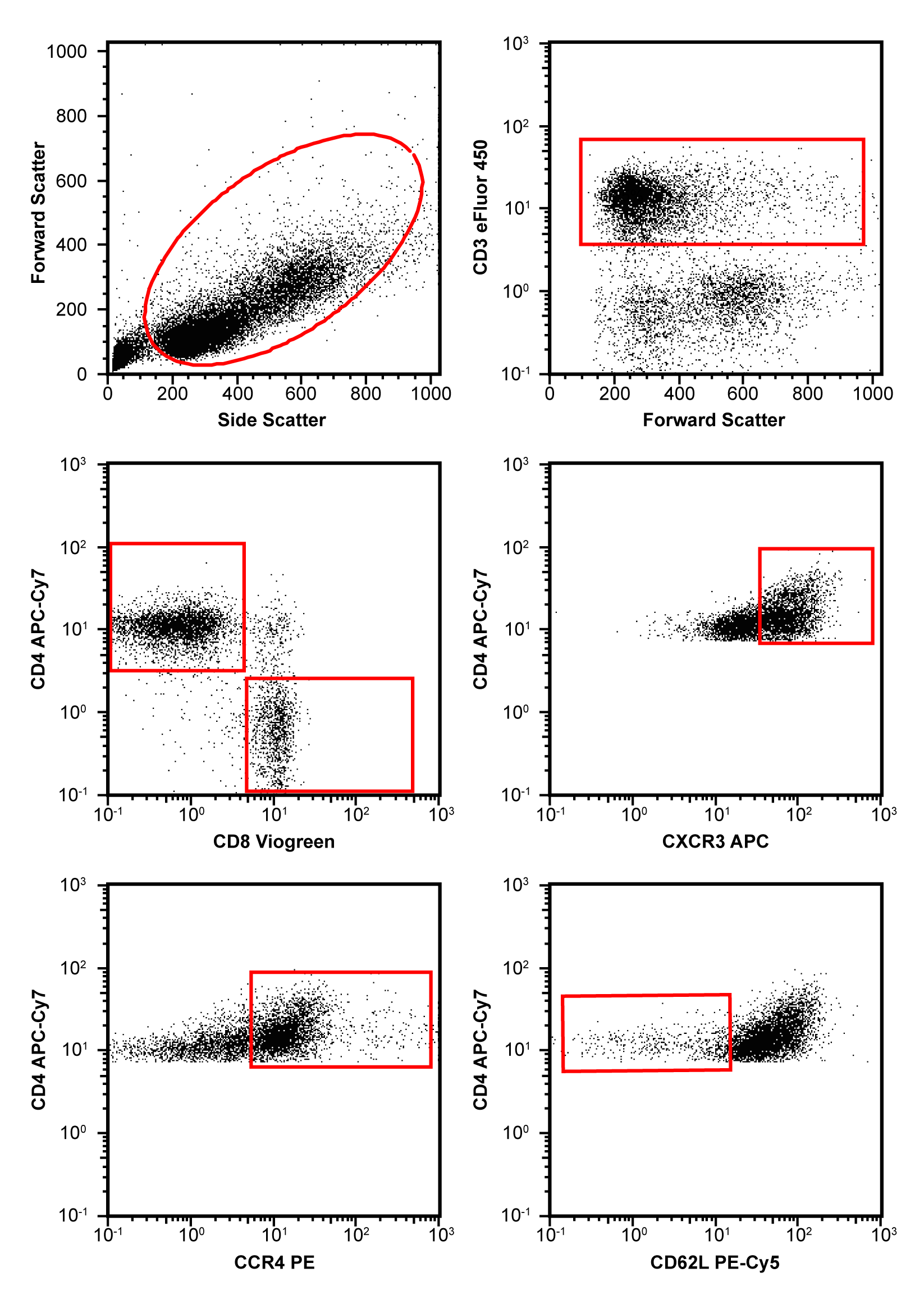

Supplement: S1 Fig — After gating on live cells using a FSC/SSC gate, a CD3 vs. FSC plot was used to determine the percentage of CD3+ T lymphocytes. Within the CD3+ population, the percentages of CD4+ helper T lymphocytes and CD8+ cytotoxic T lymphocytes were determined, before the percentages of CXCR3+ Th1 and CCR4+ Th2 lymphocytes within the CD4+ population were determined. Activation levels of T lymphocytes based on CD62Llow expression were also determined. (TIF) [file pone.0141793.s001.tif]

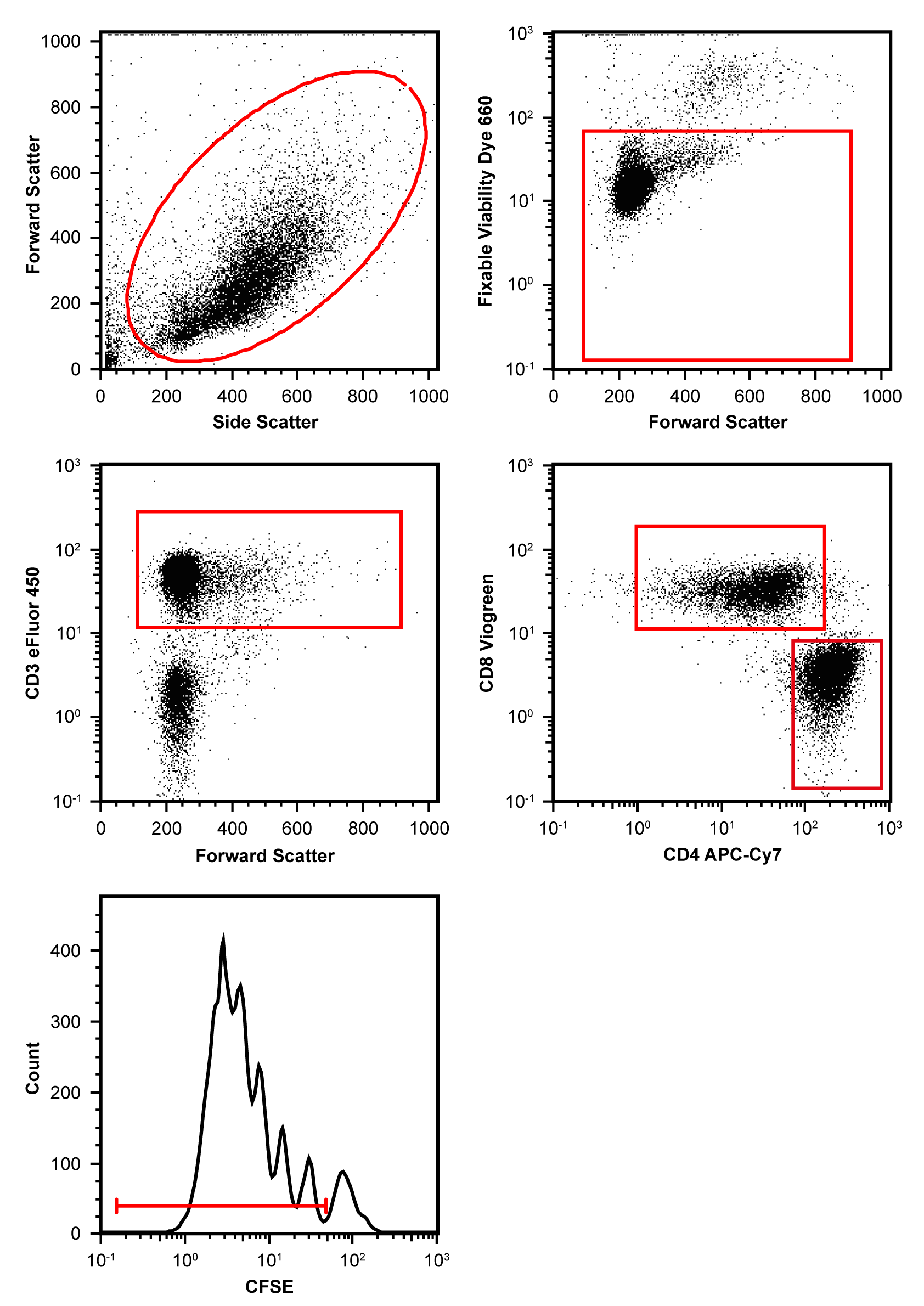

Supplement: S2 Fig — After gating on live cells using a FSC/SSC gate, non-viable cells were excluded using Fixable Viability Dye 660. A CD3 vs. FSC plot was then used to determine the percentage of CD3+ T lymphocytes. Within the CD3+ population, the percentages of CD4+ helper T lymphocytes and CD8+ cytotoxic T lymphocytes were determined before a histogram was used to analyse the CFSE fluorescence profile of each cell population. The fraction diluted statistic was calculated by creating a univariate gate below the undivided peak, while all other proliferation statistics were calculated using the proliferation analysis tool included in the FlowJo software. (TIF) [file pone.0141793.s002.tif]

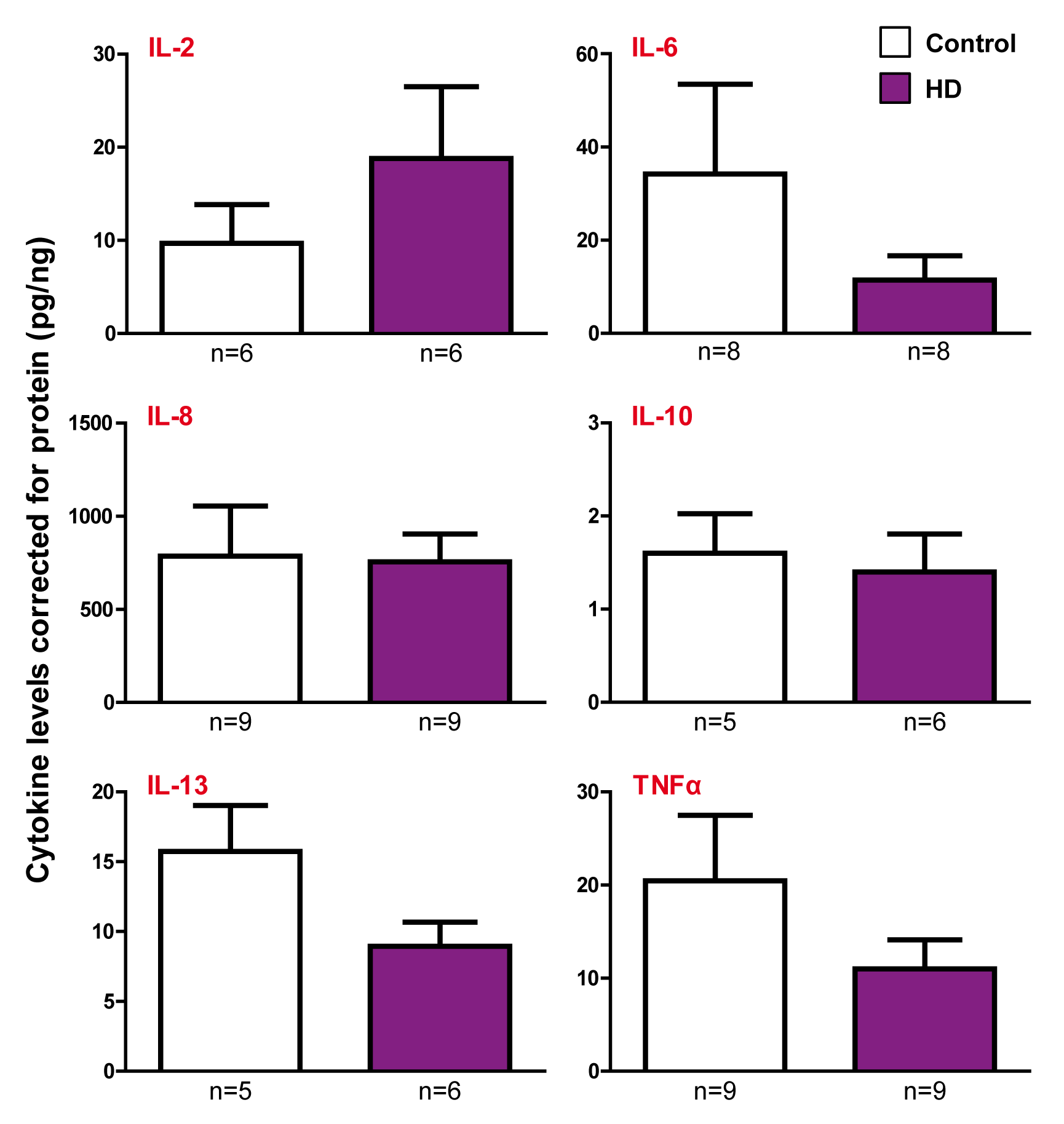

Supplement: S3 Fig — CD4+ helper T lymphocytes were isolated from HD and control peripheral blood using magnetic cell sorting and seeded without stimulation. Supernatants were collected after 48 h and cytokine profiling was carried out. No significant differences were seen in the detectable levels of any cytokines produced by HD and control CD4+ T lymphocytes after normalisation to total protein levels. Data show mean concentrations ± SEM. Statistical analysis was carried out using two-tailed unpaired student’s t tests. (TIF) [file pone.0141793.s003.tif]
