# Supplementary material for: Mutant Huntingtin Does Not Affect the Intrinsic Phenotype of Human Huntington’s Disease T Lymphocytes
Source: PLoS One. 2015 Nov 3;10(11):e0141793. doi: 10.1371/journal.pone.0141793 (PMC4631523; doi:10.1371/journal.pone.0141793)
Supplement: S1 Table — The cohort used for each experiment is listed separately. (DOCX) [file pone.0141793.s004.docx]

| **Experiment** | **Subject group** | **N** | **age**  **(mean ± SD)** | **CAG**  **(mean ± SD)** |
| --- | --- | --- | --- | --- |
| T cell subset analysis – T_h_1 cells (Fig 1 and 2) | Control | 5 | 44.9 ± 9.4 | - |
|  | HD | 7 | 52.2 ± 10.0 | 42.3 ± 1.0 |
| T cell subset analysis – all other cell types (Fig 1 and 2) | Control | 9 | 46.2 ± 12.1 | - |
|  | HD | 10 | 55.6 ± 9.9 | 42.0 ± 1.6 |
| T cell proliferation assays (Fig 3 and 4, S3-6 Tables) | Control | 9 | 54.8 ± 7.4 | - |
|  | HD | 8 | 56.2 ± 8.6 | 43.0 ± 2.4 |
| Stimulated T cell cytokine profiling – IL-1β (Fig 5) | Control | 9 | 55.4 ± 12.6 | - |
|  | HD | 9 | 57.4 ± 11.4 | 43.2 ± 1.9 |
| Stimulated T cell cytokine profiling – IL-12p70 (Fig 5) | Control | 8 | 56.1 ± 13.3 | - |
|  | HD | 9 | 57.4 ± 11.4 | 43.2 ± 1.9 |
| Stimulated T cell cytokine profiling – all other cytokines (Fig 5) | Control | 10 | 56.4 ± 12.3 | - |
|  | HD | 9 | 57.4 ± 11.4 | 43.2 ± 1.9 |
| Unstimulated T cell cytokine profiling – IL-2 (S3 Fig) | Control | 6 | 53.9 ± 15.3 | - |
|  | HD | 6 | 52.4 ± 10.2 | 44.0 ± 1.8 |
| Unstimulated T cell cytokine profiling – IL-6 (S3 Fig) | Control | 8 | 57.1 ± 12.4 | - |
|  | HD | 8 | 57.8 ± 12.1 | 43.0 ± 1.93 |
| Unstimulated T cell cytokine profiling – IL-8 (S3 Fig) | Control | 9 | 55.1 ± 12.3 | - |
|  | HD | 9 | 57.4 ± 11.4 | 43.2 ± 1.9 |
| Unstimulated T cell cytokine profiling – IL-10 (S3 Fig) | Control | 5 | 57.7 ± 15.4 | - |
|  | HD | 6 | 54.7 ± 11.9 | 43.5 ± 2.0 |
| Unstimulated T cell cytokine profiling – IL-13 (S3 Fig) | Control | 5 | 57.1 ± 15.5 | - |
|  | HD | 6 | 56.9 ± 14.2 | 43.5 ± 2.0 |
| Unstimulated T cell cytokine profiling – TNFα (S3 Fig) | Control | 9 | 55.4 ± 12.6 | - |
|  | HD | 9 | 57.4 ± 11.4 | 43.2 ± 1.9 |
| PCR arrays (Tables 1 and 2, S7 and S8 tables) | Control | 12 | 49.3 ± 15.3 | - |
|  | HD | 11 | 58.7 ± 5.7 | 42.6 ± 1.4 |
