# Supplementary material for: Mutant Huntingtin Does Not Affect the Intrinsic Phenotype of Human Huntington’s Disease T Lymphocytes
Source: PLoS One. 2015 Nov 3;10(11):e0141793. doi: 10.1371/journal.pone.0141793 (PMC4631523; doi:10.1371/journal.pone.0141793)
Supplement: S2 Table — (DOCX) [file pone.0141793.s005.docx]

| **Antibody** | **Amount used** | **Company** |
| --- | --- | --- |
| Anti-human CD194 (CCR4) PE | 5 µl per test | BD Biosciences |
| Anti-human CD3 eFluor450 | 2 µl per test | eBioscience |
| Anti-human CD4 APC-Cy7 | 2 µl per test | BD Biosciences |
| Anti-human CD8 VioGreen | 4.5 µl per test | Miltenyi Biotec |
| Anti-human CD62L PE-Cy5 | 5 µl per test | eBioscience |
| Anti-human CXCR3 APC | 5 µl per test | BioLegend |
