# Supplementary material for: Mutant Huntingtin Does Not Affect the Intrinsic Phenotype of Human Huntington’s Disease T Lymphocytes
Source: PLoS One. 2015 Nov 3;10(11):e0141793. doi: 10.1371/journal.pone.0141793 (PMC4631523; doi:10.1371/journal.pone.0141793)
Supplement: S3 Table — Percentage divided is calculated as the percentage of cells from the initial culture which divided at least once. HD n = 8, control n = 9. Data shown as mean ± SEM. (DOCX) [file pone.0141793.s006.docx]

| **Cell type** | **Treatment** | **Time point** | **Percentage divided** | |
| --- | --- | --- | --- | --- |
|  |  |  | **Control** | **HD** |
| T lymphocytes  (CD3^+^) | Unstimulated | 72 h | 0.32 ± 0.08 | 0.31 ± 0.07 |
|  | Unstimulated | 96 h | 1.12 ± 0.22 | 1.36 ± 0.42 |
|  | Unstimulated | 120 h | 1.05 ± 0.22 | 1.06 ± 0.26 |
|  | Anti-CD3 + CD28 | 72 h | 42.68 ± 6.91 | 48.01 ± 6.84 |
|  | Anti-CD3 + CD28 | 96 h | 59.73 ± 6.37 | 51.78 ± 9.02 |
|  | Anti-CD3 + CD28 | 120 h | 60.80 ± 6.70 | 65.09 ± 6.39 |
|  | PHA-P | 72 h | 53.01 ± 4.95 | 40.02 ± 7.24 |
|  | PHA-P | 96 h | 54.17 ± 4.63 | 43.98 ± 7.26 |
|  | PHA-P | 120 h | 52.58 ± 3.27 | 39.11 ± 6.51 |
| Helper T lymphocytes (CD3^+^ CD4^+^) | Unstimulated | 72 h | 0.20 ± 0.08 | 0.28 ± 0.08 |
|  | Unstimulated | 96 h | 0.96 ± 0.18 | 1.11 ± 0.49 |
|  | Unstimulated | 120 h | 1.11 ± 0.31 | 1.18 ± 0.49 |
|  | Anti-CD3 + CD28 | 72 h | 46.24 ± 5.56 | 54.71 ± 5.85 |
|  | Anti-CD3 + CD28 | 96 h | 64.98 ± 5.03 | 58.96 ± 9.53 |
|  | Anti-CD3 + CD28 | 120 h | 67.44 ± 4.04 | 79.15 ± 5.27 |
|  | PHA-P | 72 h | 52.19 ± 5.59 | 47.95 ± 4.21 |
|  | PHA-P | 96 h | 58.91 ± 2.91 | 53.95 ± 3.72 |
|  | PHA-P | 120 h | 58.90 ± 2.21 | 50.10 ± 2.84 |
| Cytotoxic T lymphocytes (CD3^+^ CD8^+^) | Unstimulated | 72 h | 0.78 ± 0.32 | 0.61 ± 0.17 |
|  | Unstimulated | 96 h | 1.51 ± 0.40 | 1.55 ± 0.51 |
|  | Unstimulated | 120 h | 1.29 ± 0.19 | 1.58 ± 0.47 |
|  | Anti-CD3 + CD28 | 72 h | 52.86 ± 7.61 | 49.65 ± 7.10 |
|  | Anti-CD3 + CD28 | 96 h | 63.10 ± 8.72 | 57.48 ± 9.04 |
|  | Anti-CD3 + CD28 | 120 h | 63.90 ± 9.16 | 67.33 ± 8.27 |
|  | PHA-P | 72 h | 53.14 ± 9.93 | 44.92 ± 9.07 |
|  | PHA-P | 96 h | 56.00 ± 8.66 | 47.10 ± 10.22 |
|  | PHA-P | 120 h | 52.66 ± 7.94 | 44.17 ± 9.67 |
