# Supplementary material for: Mutant Huntingtin Does Not Affect the Intrinsic Phenotype of Human Huntington’s Disease T Lymphocytes
Source: PLoS One. 2015 Nov 3;10(11):e0141793. doi: 10.1371/journal.pone.0141793 (PMC4631523; doi:10.1371/journal.pone.0141793)
Supplement: S4 Table — Proliferation index is calculated as the average number of divisions undergone by the dividing cells only. HD n = 8, control n = 9. Data shown as mean ± SEM. (DOCX) [file pone.0141793.s007.docx]

| **Cell type** | **Treatment** | **Time point** | **Proliferation index** | |
| --- | --- | --- | --- | --- |
|  |  |  | **Control** | **HD** |
| T lymphocytes  (CD3^+^) | Unstimulated | 72 h | 1.25 ± 0.04 | 1.39 ± 0.14 |
|  | Unstimulated | 96 h | 1.60 ± 0.06 | 1.47 ± 0.08 |
|  | Unstimulated | 120 h | 2.13 ± 0.05 | 1.75 ± 0.17 |
|  | Anti-CD3 + CD28 | 72 h | 1.59 ± 0.09 | 1.62 ± 0.08 |
|  | Anti-CD3 + CD28 | 96 h | 2.33 ± 0.13 | 2.49 ± 0.15 |
|  | Anti-CD3 + CD28 | 120 h | 3.27 ± 0.16 | 3.51 ± 0.20 |
|  | PHA-P | 72 h | 1.58 ± 0.06 | 1.58 ± 0.05 |
|  | PHA-P | 96 h | 2.19 ± 0.08 | 2.07 ± 0.12 |
|  | PHA-P | 120 h | 2.61 ± 0.16 | 2.30 ± 0.14 |
| Helper T lymphocytes (CD3^+^ CD4^+^) | Unstimulated | 72 h | 1.22 ± 0.04 | 1.42 ± 0.15 |
|  | Unstimulated | 96 h | 1.49 ± 0.05 | 1.48 ± 0.07 |
|  | Unstimulated | 120 h | 2.04 ± 0.06 | 1.76 ± 0.13 |
|  | Anti-CD3 + CD28 | 72 h | 1.49 ± 0.07 | 1.61 ± 0.07 |
|  | Anti-CD3 + CD28 | 96 h | 2.09 ± 0.12 | 2.43 ± 0.16 |
|  | Anti-CD3 + CD28 | 120 h | 3.05 ± 0.15 | 3.33 ± 0.23 |
|  | PHA-P | 72 h | 1.42 ± 0.04 | 1.45 ± 0.05 |
|  | PHA-P | 96 h | 1.94 ± 0.05 | 1.88 ± 0.11 |
|  | PHA-P | 120 h | 2.22 ± 0.15 | 2.06 ± 0.11 |
| Cytotoxic T lymphocytes (CD3^+^ CD8^+^) | Unstimulated | 72 h | 1.49 ± 0.24 | 1.99 ± 0.55 |
|  | Unstimulated | 96 h | 1.94 ± 0.11 | 1.69 ± 0.13 |
|  | Unstimulated | 120 h | 2.31 ± 0.24 | 1.86 ± 0.23 |
|  | Anti-CD3 + CD28 | 72 h | 1.88 ± 0.13 | 1.81 ± 0.18 |
|  | Anti-CD3 + CD28 | 96 h | 2.80 ± 0.17 | 3.00 ± 0.27 |
|  | Anti-CD3 + CD28 | 120 h | 3.88 ± 0.19 | 3.65 ± 0.27 |
|  | PHA-P | 72 h | 2.04 ± 0.11 | 1.79 ± 0.13 |
|  | PHA-P | 96 h | 2.84 ± 0.15 | 2.44 ± 0.22 |
|  | PHA-P | 120 h | 3.32 ± 0.22 | 2.76 ± 0.30 |
