# Supplementary material for: Mutant Huntingtin Does Not Affect the Intrinsic Phenotype of Human Huntington’s Disease T Lymphocytes
Source: PLoS One. 2015 Nov 3;10(11):e0141793. doi: 10.1371/journal.pone.0141793 (PMC4631523; doi:10.1371/journal.pone.0141793)
Supplement: S5 Table — Division index is calculated as the average number of divisions undergone by all cells in culture. HD n = 8, control n = 9. Data shown as mean ± SEM. (DOCX) [file pone.0141793.s008.docx]

| **Cell type** | **Treatment** | **Time point** | **Division index** | |
| --- | --- | --- | --- | --- |
|  |  |  | **Control** | **HD** |
| T lymphocytes  (CD3^+^) | Unstimulated | 72 h | 0.004 ± 0.001 | 0.005 ± 0.001 |
|  | Unstimulated | 96 h | 0.018 ± 0.004 | 0.020 ± 0.006 |
|  | Unstimulated | 120 h | 0.022 ± 0.004 | 0.020 ± 0.006 |
|  | Anti-CD3 + CD28 | 72 h | 0.72 ± 0.15 | 0.81 ± 0.16 |
|  | Anti-CD3 + CD28 | 96 h | 1.44 ± 0.20 | 1.35 ± 0.28 |
|  | Anti-CD3 + CD28 | 120 h | 2.13 ± 0.31 | 2.31 ± 0.27 |
|  | PHA-P | 72 h | 0.86 ± 0.10 | 0.63 ± 0.12 |
|  | PHA-P | 96 h | 1.21 ± 0.14 | 0.95 ± 0.18 |
|  | PHA-P | 120 h | 1.38 ± 0.13 | 0.94 ± 0.19 |
| Helper T lymphocytes (CD3^+^ CD4^+^) | Unstimulated | 72 h | 0.003 ± 0.001 | 0.004 ± 0.001 |
|  | Unstimulated | 96 h | 0.014 ± 0.003 | 0.016 ± 0.006 |
|  | Unstimulated | 120 h | 0.022 ± 0.006 | 0.021 ± 0.010 |
|  | Anti-CD3 + CD28 | 72 h | 0.71 ± 0.11 | 0.90 ± 0.13 |
|  | Anti-CD3 + CD28 | 96 h | 1.46 ± 0.16 | 1.49 ± 0.29 |
|  | Anti-CD3 + CD28 | 120 h | 2.06 ± 0.17 | 2.66 ± 0.26 |
|  | PHA-P | 72 h | 0.75 ± 0.09 | 0.69 ± 0.06 |
|  | PHA-P | 96 h | 1.15 ± 0.07 | 1.03 ± 0.11 |
|  | PHA-P | 120 h | 1.32 ± 0.12 | 1.04 ± 0.09 |
| Cytotoxic T lymphocytes (CD3^+^ CD8^+^) | Unstimulated | 72 h | 0.009 ± 0.003 | 0.011 ± 0.005 |
|  | Unstimulated | 96 h | 0.031 ± 0.010 | 0.025 ± 0.009 |
|  | Unstimulated | 120 h | 0.032 ± 0.007 | 0.032 ± 0.014 |
|  | Anti-CD3 + CD28 | 72 h | 1.06 ± 0.21 | 0.98 ± 0.23 |
|  | Anti-CD3 + CD28 | 96 h | 1.88 ± 0.33 | 1.78 ± 0.39 |
|  | Anti-CD3 + CD28 | 120 h | 2.57 ± 0.42 | 2.49 ± 0.38 |
|  | PHA-P | 72 h | 1.15 ± 0.25 | 0.87 ± 0.19 |
|  | PHA-P | 96 h | 1.66 ± 0.30 | 1.29 ± 0.30 |
|  | PHA-P | 120 h | 1.79 ± 0.33 | 1.37 ± 0.33 |
