# Supplementary material for: Mutant Huntingtin Does Not Affect the Intrinsic Phenotype of Human Huntington’s Disease T Lymphocytes
Source: PLoS One. 2015 Nov 3;10(11):e0141793. doi: 10.1371/journal.pone.0141793 (PMC4631523; doi:10.1371/journal.pone.0141793)
Supplement: S6 Table — Fraction diluted is calculated as the percentage of cells in the final culture which have divided at least once. HD n = 8, control n = 9. Data shown as mean ± SEM. (DOCX) [file pone.0141793.s009.docx]

| **Cell type** | **Treatment** | **Time point** | **Fraction diluted** | |
| --- | --- | --- | --- | --- |
|  |  |  | **Control** | **HD** |
| T lymphocytes  (CD3^+^) | Unstimulated | 72 h | 1.00 ± 0.39 | 1.69 ± 0.71 |
|  | Unstimulated | 96 h | 4.13 ± 0.85 | 3.59 ± 1.30 |
|  | Unstimulated | 120 h | 6.95 ± 0.98 | 5.46 ± 1.87 |
|  | Anti-CD3 + CD28 | 72 h | 62.17 ± 7.73 | 69.19 ± 5.96 |
|  | Anti-CD3 + CD28 | 96 h | 85.56 ± 5.14 | 79.91 ± 7.81 |
|  | Anti-CD3 + CD28 | 120 h | 93.92 ± 2.02 | 96.15 ± 1.33 |
|  | PHA-P | 72 h | 70.68 ± 4.89 | 58.05 ± 8.28 |
|  | PHA-P | 96 h | 83.71 ± 3.62 | 70.90 ± 9.25 |
|  | PHA-P | 120 h | 87.97 ± 2.70 | 76.05 ± 7.27 |
| Helper T lymphocytes (CD3^+^ CD4^+^) | Unstimulated | 72 h | 0.82 ± 0.35 | 1.26 ± 0.44 |
|  | Unstimulated | 96 h | 3.18 ± 0.52 | 3.26 ± 1.35 |
|  | Unstimulated | 120 h | 6.29 ± 1.24 | 5.24 ± 1.98 |
|  | Anti-CD3 + CD28 | 72 h | 66.54 ± 6.57 | 75.51 ± 4.64 |
|  | Anti-CD3 + CD28 | 96 h | 88.42 ± 3.99 | 83.13 ± 6.83 |
|  | Anti-CD3 + CD28 | 120 h | 95.83 ± 1.03 | 97.69 ± 1.00 |
|  | PHA-P | 72 h | 68.29 ± 6.20 | 65.89 ± 4.57 |
|  | PHA-P | 96 h | 84.99 ± 2.04 | 79.98 ± 3.65 |
|  | PHA-P | 120 h | 87.89 ± 1.86 | 84.41 ± 1.94 |
| Cytotoxic T lymphocytes (CD3^+^ CD8^+^) | Unstimulated | 72 h | 1.09 ± 0.43 | 4.36 ± 2.83 |
|  | Unstimulated | 96 h | 7.06 ± 2.17 | 4.82 ± 1.88 |
|  | Unstimulated | 120 h | 11.15 ± 2.44 | 8.29 ± 3.61 |
|  | Anti-CD3 + CD28 | 72 h | 73.59 ± 7.51 | 73.20 ± 6.03 |
|  | Anti-CD3 + CD28 | 96 h | 87.48 ± 5.56 | 85.24 ± 6.62 |
|  | Anti-CD3 + CD28 | 120 h | 93.57 ± 3.21 | 96.54 ± 1.52 |
|  | PHA-P | 72 h | 72.32 ± 7.99 | 63.76 ± 10.91 |
|  | PHA-P | 96 h | 85.54 ± 5.14 | 71.91 ± 12.91 |
|  | PHA-P | 120 h | 90.14 ± 3.69 | 75.46 ± 12.02 |
