# Supplementary material for: Mutant Huntingtin Does Not Affect the Intrinsic Phenotype of Human Huntington’s Disease T Lymphocytes
Source: PLoS One. 2015 Nov 3;10(11):e0141793. doi: 10.1371/journal.pone.0141793 (PMC4631523; doi:10.1371/journal.pone.0141793)
Supplement: S7 Table — Data presented as fold change calculated from ΔΔ-CT values, unpaired two-tailed t-test used as statistical method. (DOCX) [file pone.0141793.s010.docx]

| **Gene name** | **Fold change** | **p value** |
| --- | --- | --- |
| TNFSF4 | 1.471 | 0.008 |
| PTGDR2 | 1.788 | 0.014 |
| GATA3 | 1.177 | 0.056 |
| IL18R1 | 1.336 | 0.058 |
| IL5 | 2.245 | 0.063 |
| LAT | 0.893 | 0.101 |
| IL2RA | 1.154 | 0.102 |
| CSF2 | 0.490 | 0.124 |
| JAK1 | 1.108 | 0.137 |
| CCR4 | 1.258 | 0.183 |
| TLR6 | 1.497 | 0.198 |
| TNFRSF9 | 0.564 | 0.227 |
| IL13 | 0.689 | 0.227 |
| IL12RB2 | 0.789 | 0.255 |
| CD86 | 0.681 | 0.270 |
| IL10 | 1.580 | 0.271 |
| BCL6 | 0.737 | 0.276 |
| IL2 | 1.297 | 0.281 |
| IL15 | 0.858 | 0.296 |
| LTA | 0.905 | 0.299 |
| STAT6 | 2.548 | 0.307 |
| CREBBP | 1.089 | 0.318 |
| IL6 | 0.309 | 0.319 |
| SPP1 | 0.317 | 0.322 |
| CTLA4 | 1.082 | 0.335 |
| EBI3 | 0.409 | 0.348 |
| IL4R | 1.080 | 0.352 |
| CD40LG | 1.090 | 0.354 |
| CD28 | 1.067 | 0.369 |
| GFI1 | 1.167 | 0.370 |
| MAPK8 | 1.055 | 0.372 |
| IL18 | 0.684 | 0.375 |
| IRF1 | 0.897 | 0.388 |
| CCL7 | 0.608 | 0.391 |
| TBX21 | 1.203 | 0.400 |
| TNF | 0.856 | 0.417 |
| IFNG | 0.780 | 0.422 |
| CD4 | 1.051 | 0.452 |
| IL6R | 1.054 | 0.459 |
| LAG3 | 1.043 | 0.459 |
| FASLG | 1.106 | 0.464 |
| NFATC2 | 1.089 | 0.474 |
| NFATC1 | 0.955 | 0.512 |
| CD27 | 0.942 | 0.522 |
| IL1R1 | 1.114 | 0.527 |
| IRF4 | 0.977 | 0.537 |
| IL24 | 1.049 | 0.587 |
| SLC11A1 | 0.994 | 0.603 |
| PTPRC | 0.964 | 0.616 |
| IL7 | 1.078 | 0.671 |
| HAVCR2 | 0.839 | 0.672 |
| CCR2 | 1.116 | 0.677 |
| YY1 | 1.029 | 0.682 |
| IL7R | 0.956 | 0.710 |
| CXCR3 | 1.166 | 0.711 |
| IL27RA | 0.969 | 0.741 |
| IL13RA1 | 0.675 | 0.743 |
| JAK2 | 1.083 | 0.749 |
| CD80 | 0.899 | 0.763 |
| CCR5 | 1.031 | 0.775 |
| ICOS | 0.962 | 0.777 |
| STAT1 | 0.972 | 0.787 |
| STAT4 | 1.007 | 0.808 |
| TGFB3 | 1.137 | 0.811 |
| TYK2 | 1.013 | 0.825 |
| PCGF2 | 0.985 | 0.831 |
| VEGFA | 0.661 | 0.898 |
| SOCS1 | 0.983 | 0.911 |
| MAF | 1.052 | 0.919 |
| TLR4 | 0.855 | 0.937 |
| CCL5 | 1.146 | 0.956 |
| CCR3 | 0.961 | 0.956 |
| SOCS5 | 0.992 | 0.969 |
| CEBPB | 0.947 | 0.971 |
