# Supplementary material for: Mutant Huntingtin Does Not Affect the Intrinsic Phenotype of Human Huntington’s Disease T Lymphocytes
Source: PLoS One. 2015 Nov 3;10(11):e0141793. doi: 10.1371/journal.pone.0141793 (PMC4631523; doi:10.1371/journal.pone.0141793)
Supplement: S8 Table — Data presented as fold change calculated from ΔΔ-CT values, unpaired two-tailed t-test used as statistical method. (DOCX) [file pone.0141793.s011.docx]

| **Gene name** | **Fold change** | **p value** |
| --- | --- | --- |
| TYK2 | 1.176 | 0.002 |
| TNFRSF8 | 1.702 | 0.013 |
| STAT6 | 1.231 | 0.035 |
| TNFSF4 | 1.756 | 0.066 |
| JAK1 | 1.169 | 0.072 |
| CCR4 | 1.191 | 0.118 |
| IL18R1 | 1.129 | 0.149 |
| CD27 | 1.261 | 0.152 |
| STAT1 | 0.844 | 0.162 |
| IL15 | 0.800 | 0.184 |
| IL5 | 0.724 | 0.197 |
| TGFB3 | 1.536 | 0.211 |
| MAPK8 | 1.082 | 0.215 |
| CD28 | 1.104 | 0.237 |
| YY1 | 1.052 | 0.272 |
| GATA3 | 1.817 | 0.300 |
| IL9 | 0.704 | 0.308 |
| IL6R | 1.174 | 0.319 |
| FASLG | 1.320 | 0.319 |
| CCR5 | 1.194 | 0.327 |
| CD40LG | 1.119 | 0.336 |
| VEGFA | 1.126 | 0.348 |
| IL2RA | 1.036 | 0.350 |
| IL12RB2 | 0.712 | 0.351 |
| NFATC2 | 1.088 | 0.360 |
| TLR6 | 0.848 | 0.361 |
| EBI3 | 0.848 | 0.366 |
| SPP1 | 0.949 | 0.367 |
| IL18 | 0.539 | 0.370 |
| IL27 | 0.747 | 0.376 |
| PTGDR2 | 1.122 | 0.381 |
| IL4 | 0.743 | 0.383 |
| BCL6 | 1.080 | 0.411 |
| IL10 | 0.932 | 0.446 |
| IL27RA | 1.040 | 0.452 |
| CTLA4 | 1.082 | 0.452 |
| SLC11A1 | 0.820 | 0.458 |
| NFATC1 | 1.055 | 0.468 |
| PCGF2 | 0.899 | 0.470 |
| GFI1 | 1.095 | 0.474 |
| LAT | 0.838 | 0.500 |
| HAVCR2 | 1.040 | 0.503 |
| IL12B | 1.018 | 0.504 |
| TNFRSF9 | 1.162 | 0.506 |
| IL4R | 0.920 | 0.507 |
| CSF2 | 1.173 | 0.511 |
| IRF4 | 1.024 | 0.517 |
| CEBPB | 1.117 | 0.532 |
| IL2 | 0.858 | 0.574 |
| SOCS5 | 1.036 | 0.585 |
| STAT4 | 1.046 | 0.590 |
| CCR3 | 0.773 | 0.593 |
| JAK2 | 1.025 | 0.595 |
| IL1R1 | 0.990 | 0.626 |
| TNF | 0.987 | 0.626 |
| IL7 | 1.079 | 0.646 |
| CCR2 | 1.181 | 0.647 |
| CREBBP | 1.027 | 0.648 |
| IL6 | 0.672 | 0.666 |
| CD4 | 1.115 | 0.697 |
| LTA | 1.071 | 0.704 |
| CCL7 | 0.396 | 0.721 |
| IL1RL1 | 1.578 | 0.738 |
| CXCR3 | 0.965 | 0.738 |
| SOCS1 | 0.941 | 0.739 |
| LAG3 | 1.033 | 0.760 |
| TLR4 | 0.935 | 0.774 |
| TBX21 | 0.926 | 0.783 |
| IL3 | 0.777 | 0.783 |
| CD86 | 0.904 | 0.806 |
| IL24 | 0.959 | 0.825 |
| PTPRC | 1.003 | 0.826 |
| CD80 | 0.982 | 0.884 |
| IL13 | 0.955 | 0.920 |
| IFNG | 0.808 | 0.929 |
| IL13RA1 | 0.949 | 0.938 |
| CCL5 | 1.059 | 0.947 |
| ICOS | 1.002 | 0.953 |
| IRF1 | 0.999 | 0.976 |
| IL7R | 1.006 | 0.979 |
| MAF | 0.924 | 0.987 |
